# A rare case of Beckwith-Wiedemann syndrome with encephalocele

**DOI:** 10.11604/pamj.2020.37.317.26158

**Published:** 2020-12-07

**Authors:** Rakesh Khatana, Anamika Khatana

**Affiliations:** 1 Mahatma Gandhi Ayurved College hospital and research Centre, Datta Meghe Institute of Medical Sciences, Wardha, Maharashtra, India,; 2 Department of Rog-Nidan, Shri Dhanwantri Ayurvedic Medical College and Research Centre, Chhata, Mathura (UP)

**Keywords:** Macrosomia, macroglossia, encephalocele

## Image in medicine

A premature female child was delivered with polyhydramnios, an unusually large placenta and long umbilical cord. The clinical examination revealed that there is macrosomia, macroglossia and encephalocele. The patient also had complains of hypoglycemia on 2^nd^ day and on the clinical examination, the patient was diagnosed with a rare case of Beckwith-Wiedemann Syndrome with encephalocele.

**Figure 1:**
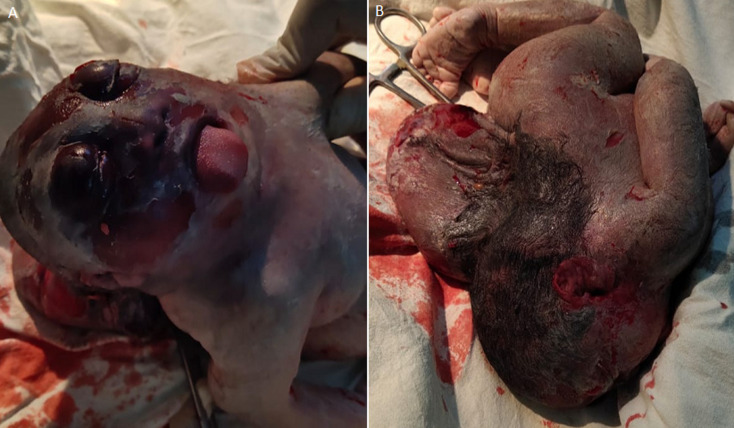
A) anterior view with macroglossia and macrosomia; B) posterior view with encephalocele

